# Effect of one session of hyperbaric oxygen (2.5 ATA for 60 min) after moderate-intensity exercise on fatigue: a single-blind crossover randomized trial

**DOI:** 10.3389/fspor.2025.1690794

**Published:** 2025-11-13

**Authors:** Kazuyoshi Yagishita, Junya Aizawa, Shunske Ohji, Takashi Hoshino, Takuya Oyaizu

**Affiliations:** 1 Hyperbaric Medical Center, Science Tokyo Hospital, Institute of Science Tokyo, Tokyo, Japan; 2 Sports Science Department, Science Tokyo Hospital, Institute of Science Tokyo, Tokyo, Japan; 3 Department of Physical Therapy, Juntendo University, Tokyo, Japan; 4 Department of Orthopaedic Surgery, Institute of Science Tokyo, Tokyo, Japan

**Keywords:** hyperbaric oxygen, fatigue, moderate-intensity exercise, randomized trial, visual analog scale (VAS)

## Abstract

**Objectives:**

Although insufficient delivery of oxygen may be a factor in physical and perceived fatigue, the relationship between exposure to hyperbaric oxygen (HBO) and recovery from perceived fatigue remains unexplained. The purpose of this single-blind study was to investigate the effects of exposure to HBO after long-duration, medium-intensity training on recovery from perceived fatigue.

**Methods:**

Fatigue was induced in nine male university students (mean age: 21.3 years) using an ergometer exercise bike at a moderate intensity of 75% of their maximum heart rate for 60 min. Post-workout, the subjects randomly received an intervention comprising exposure to HBO or mild hyperbaric air (MHA) as a control in a single-blind experimental trial. Blood tests were conducted, and perceived fatigue was evaluated using the visual analog scale (VAS) at five time points. A crossover trial was conducted 1 week later.

**Results:**

In the HBO group, the pre- to post-intervention mean VAS scores for whole-body fatigue significantly improved from 48.4 to 28.7 (*p* < 0.001). However, in the MHA group, the improvement was not statistically significant. The majority of the hematological assessments, including C-reactive protein level, white blood cell count, creatine kinase level, lactic acid level, T-cell count, CD4/CD8 ratio, and natural killer cell count, did not show any significant between-group differences; however, the blood urea nitrogen and free fatty acids levels 1.5 h after intervention and Mg levels immediately after intervention were significantly higher in the HBO group.

**Conclusions:**

The results of this study showed that perceived fatigue evaluated using the VAS score significantly improved in the HBO group in a blinded trial. However, this was not objectively supported by the blood test results. HBO may have effects on recovery from perceived fatigue following long-duration, moderate-intensity exercise; however, the results of this study could not determine the efficacy of HBO on exercise-induced fatigue.

**Clinical Trial Registration:**

https://www.isrctn.com/ISRCTN81080077, identifier ISRCTN81080077.

## Introduction

Hyperbaric oxygen (HBO) treatment is an intervention in which an individual breathes near 100% oxygen intermittently while inside a hyperbaric chamber that is pressurized to greater than the sea level pressure [1 atmosphere absolute (ATA)]. For clinical purposes, the pressure must be equal to or exceed 1.4 ATA while breathing near 100% oxygen (1). HBO can increase serum dissolved oxygen levels in proportion to oxygen partial pressure and transport dissolved oxygen to ischemic tissue. HBO is an established treatment for several conditions, such as decompression illness, carbon monoxide poisoning, arterial gas embolism, peripheral ischemic disease, compartment syndrome, and delayed radiation injury.

The effects of HBO treatment on soft tissue injuries during sports activities, including sprains (2, 3), ligament injuries (4–7), contusions, and muscle strains (8–13), have been widely reported. HBO is an effective treatment for edema reduction and improves local perfusion and oxygenation of the injured tissues (3, 13–15).

However, there is disagreement regarding the effectiveness of HBO treatment in promoting recovery from exercise-induced fatigue. Studies have suggested that it has a positive effect, as a study has shown that HBO treatment for delayed onset muscle soreness (DOMS) resulting from eccentric training of the quadriceps promoted eccentric torque recovery (16). Furthermore, more recently, a study on the effects of HBO treatment on muscle fatigue after repeated plantar flexion of the ankle joint found that HBO treatment reduced the decline in muscle force production (17). Moreover, in a study among Brazilian jiu-jitsu athletes, although no HBO intervention-related differences were found in the post-training blood tests, a significant difference in perceived recovery scale scores was found, suggesting an effect on self-perceived recovery (18). Finally, the insufficient delivery of oxygen has also been shown to be a factor in the development of central and peripheral fatigue (19).

In contrast, studies have also found that HBO treatment results in no significant difference in recovery from DOMS caused by eccentric workouts of the elbow or knee flexor muscle groups (20–22). Further, a Cochrane Review of research on the effectiveness of HBO for DOMS and closed soft tissue injury treatment found that the evidence was insufficient to establish its superiority over other treatments (12). Therefore, studies that are carefully designed for validity are needed to establish the effectiveness of HBO treatment for fatigue. Thus, this study was designed as a single-blind, crossover randomized study with detailed multiple-biomarker items.

We hypothesized that HBO would reduce fatigue after long-duration, moderate-intensity exercise. The purpose of this single-blind crossover randomized study was to evaluate the effectiveness of HBO treatment for fatigue after exercise in healthy male university students who regularly exercise.

## Materials and methods

### Trial design

This study used a crossover design in which all the subjects received both the HBO and MHA interventions following a training session with an interval of 1 week. In the first trial, they were randomly assigned to either the HBO or MHA group. Randomization was conducted (KY) by rolling a die, and the participants were registered in a particular group if they rolled an odd or even number (TO). Only the researchers knew the treatment that each subject was receiving, making it a single-blind study. The validity of the study design was ensured by using a single-blind crossover randomized trial and tests for physical and mental effects using blood tests and self-rated evaluation scales.

### Participants

The subjects were healthy male university students who exercised regularly. To be eligible, the students needed to be at least ≥20 and <30 years of age at the time they gave their informed consent. Receipt of written consent was considered an indication that the student had voluntarily agreed to participate after receiving and understanding a thorough explanation of the study, and written informed consent was obtained from all the subjects prior to participation. Eligible students were eliminated from the sample if they had difficulty relieving pressure in their ears (which precludes or makes HBO treatment difficult), claustrophobia, congenital pulmonary cysts, asthma, a history of pneumothorax or heart disease, or if they had experienced heart palpitations, precordial pain, or tachycardia during the previous year.

### Sample size calculations

The sample size was calculated to be 15 people based on an alpha error of 0.05, a detection rate of 0.8, and an effect size of 0.5. In the protocol for the ethics review, there were 15 people in each of the HBO, oxygen, and air groups. However, the oxygen group was not implemented, and the study was changed to a crossover study of the HBO group and the air group. In reality, nine students were recruited. None of the eligible students met any of the exclusion criteria. The data were collected at Tokyo Medical and Dental University Hospital.

### Exercise protocol

Fatigue was induced by having the subjects ride an ergometer exercise bike (Aerobike 75XL, COMBI Wellness Corp., Tokyo, Japan) at a medium intensity of 75% of their maximum heart rate, with a target pulse rate of 150 per minute for 60 min.

### HBO and MHA interventions

**Figure 1:**
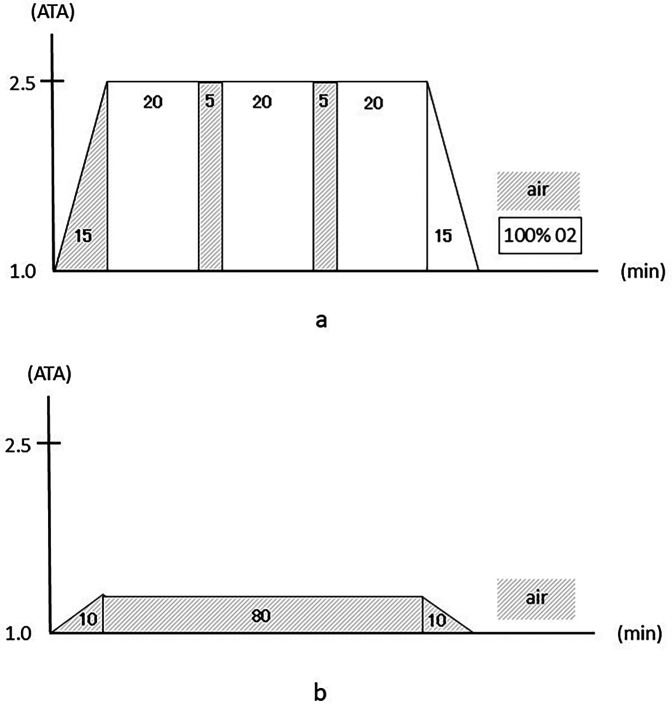
**(a)** The hyperbaric oxygen intervention protocol. **(b)** The mild hyperbaric air intervention protocol.

The HBO chamber in our university hospital was used; this multiplace HBO chamber can hold a maximum of 16 individuals (NHC-412-A, Nakamura Tekko-Sho Corp., Tokyo, Japan). The HBO treatment protocol included 60 min of inhaling pure oxygen using a mask at pressures up to 2.5 ATA with two 5-min breaks to breathe air, 15 min for compression, and 15 min for decompression, for a total of 100 min (Figure 1a). This treatment protocol is the standard for HBO treatment and is used globally.

For the MHA protocol as a control, we chose a pressure and time duration that were safe enough to prevent decompression sickness. The intervention consisted of 80 min of breathing air at pressures up to 1.2 ATA, with 10 min for compression and 10 min for decompression, for a total of 100 min (Figure 1b) (23).

To ensure that the subjects did not know whether they were being treated with HBO or air, the pressure gauges in the treatment room were hidden from their view and covered with a piece of cloth, and the tubes delivering oxygen and air were also kept out of sight.

In addition, to investigate the reliability of this single-blind study, after the subjects received the intervention, they were asked whether they thought they had received the HBO or MHA intervention. The question was asked of the nine subjects a total of 18 times. One response from three subjects each was invalid. The remaining 15 valid responses were used to determine the correct response rate.

### Measures

1. Visual analog scale (VAS)

The VAS was used to measure the participants’ perceived fatigue in their whole body and legs. The VAS score totals 100 points, with the worst condition designated as 100 points and no complaints as 0 points.

1. Blood tests

Complete blood count: white blood cells (WBCs), WBC differential, red blood cells, hemoglobin, hematocrit, and platelets.

Biochemical tests: C-reactive protein, erythrocyte sedimentation rate, aspartate transaminase, alanine transaminase, zinc tolerance test, alkaline phosphatase, lactate dehydrogenase (LDH), *γ*-glutamyl transpeptidase, cholinesterase, creatinine kinase (CK), total protein, creatinine, blood urea nitrogen (BUN), uric acid, triglycerides, total cholesterol, amylase, glucose, free fatty acids, albumin, lactate, bilirubin, potassium (K), calcium, magnesium (Mg), and myoglobin.

Immune system tests: interleukin-1β (IL-1β), IL-6, tumor necrosis factor-α (TNF-α), CD4, CD8, and natural killer (NK) cell count.

For the blood test items, a complete blood count was used as a general test, and biomarkers related to exercise and muscle function, including CK, Mg, and myoglobin, and inflammatory markers that increase immediately after exercise, such as IL-6, CD4, CD8, NK cells, and markers that change due to HBO, were selected.

### Measurement timing

**Figure 2:**
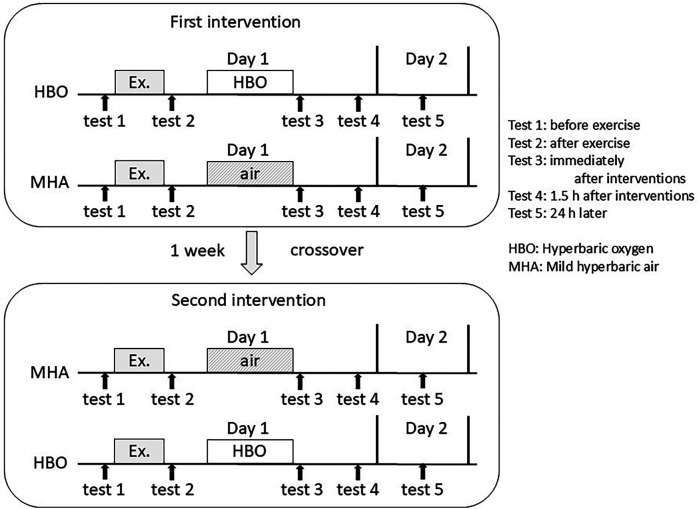
Measurement timing in this crossover trial: test 1, before exercise; test 2, after exercise; test 3, immediately after the interventions; test 4, 1.5 h after the interventions; and test 5, 24 h after the interventions.

The blood tests and VAS scores for fatigue were measured pre-exercise (test 1), post-exercise (test 2), post-intervention (test 3), 1.5 h post-intervention (test 4), and 24 h post-intervention (test 5). Test 1 was conducted 5 min before the start of exercise, test 2 was conducted 5 min after the end of exercise, and test 3 was conducted 5 min after the end of the HBO or air intervention. The interval between the end of exercise and the start of the HBO or MHA intervention was set at 20 min. The first and second trials (crossover trial) were conducted 1 week apart (Figure 2). Since myoglobin, an important marker of muscle injury, peaks more than 3 h after exercise, test 4 was scheduled 1.5 h after the intervention.

In the initial trial, five subjects received the HBO treatment and four received MHA. Subsequently, in the crossover trial, four subjects received the HBO treatment and five received MHA.

### Statistical analysis

The data are expressed as mean ± standard deviation and were analyzed using SPSS version 19.0 (SPSS Inc., Chicago, IL, USA). The VAS scores and laboratory data of the HBO and air groups were evaluated using two-way analysis of variance followed by the Bonferroni *post hoc* test, and the between-test data were evaluated using a paired *t*-test. A *p-*value < 0.05 was considered statistically significant.

## Results

### Baseline

All nine subjects completed the experiment. Their mean age was 21.3 ± 1.2 years (20‒23 years), mean height was 172.2 ± 6.2 cm, and mean weight was 82.3 ± 15.7 kg.

### Reliability of this single-blind study

**Figure 3:**
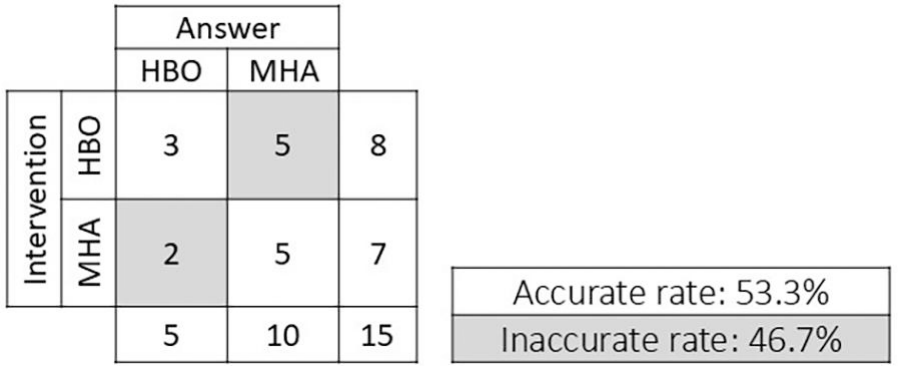
Assessment of the reliability of the blinded intervention.

Of the eight subjects who received the HBO intervention, three answered the question regarding which intervention they received correctly and five answered incorrectly with “air.” Of the seven subjects who received the MHA, five answered the same question correctly and two answered incorrectly with “HBO.” The accuracy rate was 53.3%, indicating the high reliability of this blinded trial (Figure 3).

### Side effects

No side effects were observed.

### VAS evaluation

**Figure 4:**
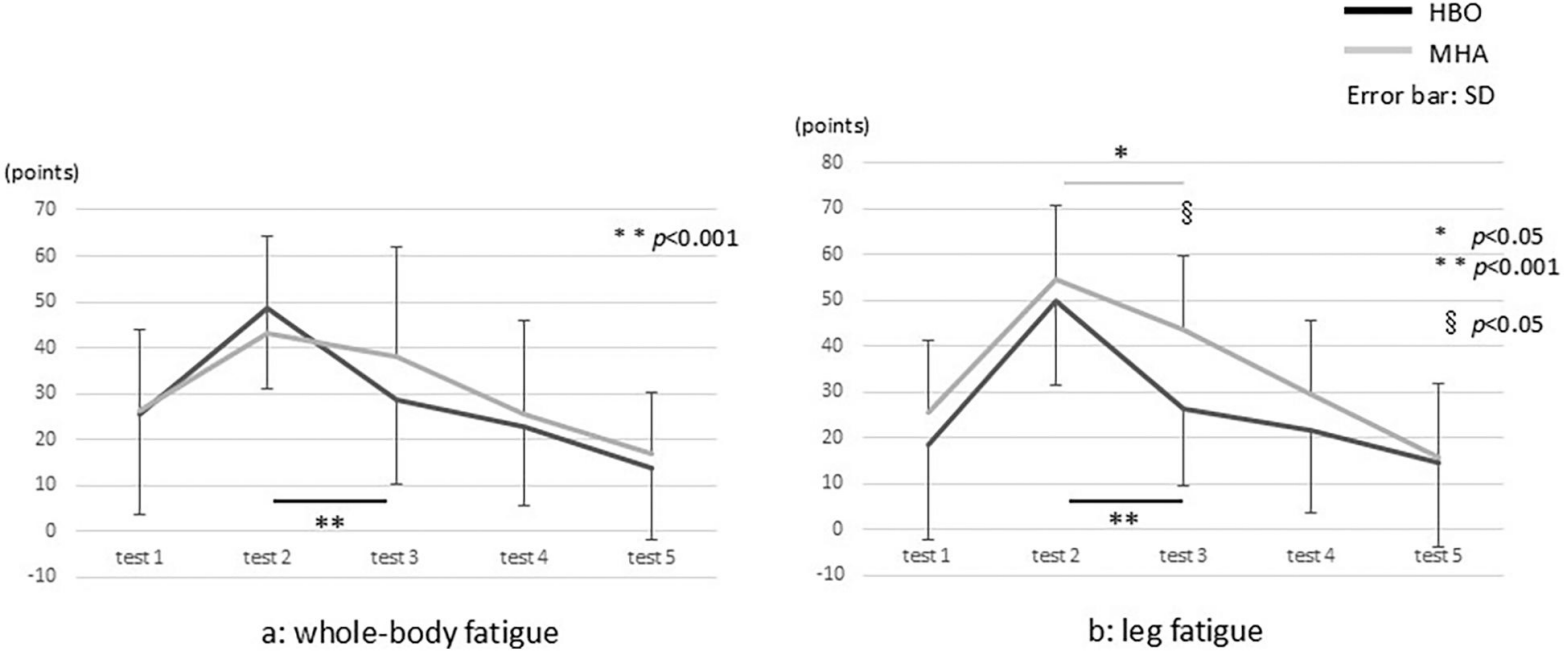
The visual analog scale results for **(a)** whole-body and **(b)** leg fatigue. * and ** indicate the comparisons between the tests, and § indicates the comparison between the groups.

Regarding the VAS scores (Figures 4a,b), in the HBO group, the mean scores for whole-body fatigue significantly decreased from pre- to post-intervention (tests 2 and 3, respectively) from 48.4 ± 17.3 to 28.8 ± 18.6 (*p* < 0.001). In the air group, the decrease was from 43.2 ± 21.2 to 37.9 ± 24.1, which was not statistically significant (Figure 4a). There were no significant differences in the data between the HBO and air groups. For leg fatigue, in the HBO group, the mean scores significantly decreased from pre- to post-intervention (tests 2 and 3, respectively) from 49.9 ± 18.4 to 26.1 ± 16.9 (*p* < 0.001). In the air group, these significantly decreased from 54.7 ± 17.1 to 43.6 ± 24.0 (*p* < 0.05) (Figure 4b). Moreover, a significant difference in the test 3 data was found between the HBO and air groups (*p* < 0.05).

### Blood test

**Figure 5:**
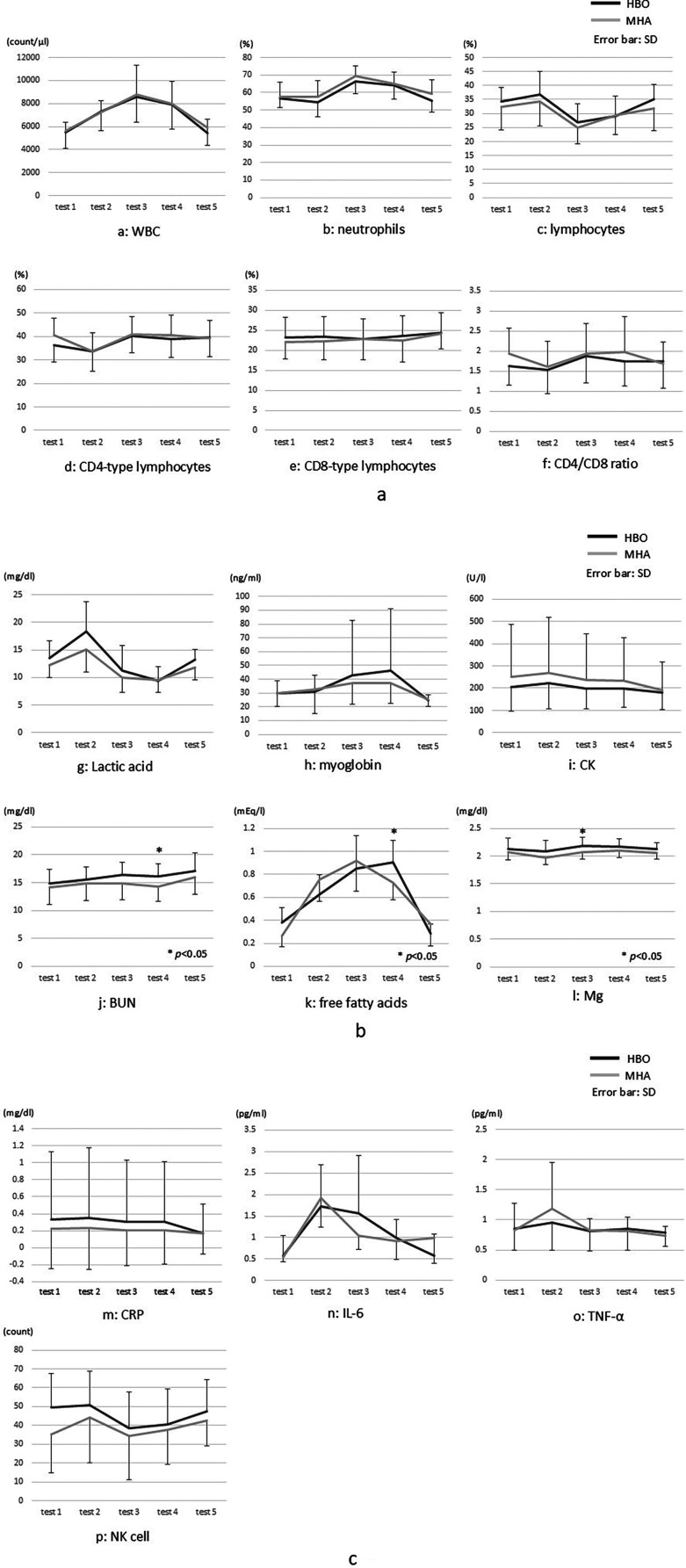
**(a–p)** Blood test results. CK, creatinine kinase; CRP, C-reactive protein; IL, interleukin; Mg, magnesium; NK, natural killer cells; TNF*α*, tumor necrosis factor alpha; WBC, white blood cells. * indicates a comparison between the groups.

The comparative analysis of the blood test results (Figure 5) between the two groups showed significantly higher BUN and free fatty acids levels in test 4 (*p* < 0.05) and Mg levels in test 3 (*p* < 0.05) in the HBO group. No other significant differences in any of the results, including lactate, were found.

The test results indicated comparatively large post-exercise changes in both groups for WBC, CD4-type lymphocyte, lactic acid, free fatty acids, and IL-6 levels in particular. WBC count increased post-exercise in both groups but returned to pre-exercise levels on the following day. Neutrophil count increased and lymphocyte count decreased in test 3, but there was no significant difference between the groups.

In the HBO group, the CD4/CD8 ratio was significantly higher in test 3 than in test 2. Although lactic acid levels markedly increased post-exercise, there was no significant difference at any time point between the groups. In the comparison of myoglobin levels from tests 2 and 3, no significant difference was found in the HBO group, and there was no significant difference at any time point between the groups.

## Discussion

### Study design and establishing the fatigue-induced workout

This study was a well-designed, single-blind, crossover randomized trial, with high reliability given a response accuracy rate of 53.3% and an expected value of 50.0%.

In the review of studies on the effects of HBO treatment on recovery from fatigue after exercise, only two crossover studies have been conducted, and there were no randomized studies (23). In addition, establishing a protocol for a control group's use of a hyperbaric chamber is generally difficult. At the very least, all the groups need to be inside the chamber for the same amount of time. In this study, similar to the approach of Babul et al., the controls (air group) breathed air (21% oxygen) through a mask at a pressure of 1.2 ATA (2). According to a review of control group protocols in single-blind HBO treatment studies, rather than using the same pressure as in the HBO treatment, it is more valid to have the controls breathe air consisting of 21% oxygen at a lower ATA than for the HBO treatment (24). In the present study, when the subjects were asked whether they had been breathing HBO or air at 1.2 ATA, the correct response rate was 53.3%. Thus, approximately 50% were mistaken, suggesting that the protocol for the control group was valid.

HBO studies have used various types of workouts to induce fatigue. A recent study using jiu-jitsu training sessions set the workout time and intensity at 1.5 h and the highest possible intensity (9). While some previous studies have used arm exercises (19), in this study, because a workout was needed that would affect the whole body rather than only specific muscles, we decided to have the subjects train for a long time at a moderate intensity until exhaustion using an ergometer exercise bike.

Changes in lymphocyte count can be used as an indicator of exercise intensity because the count increases until the subject finishes exercising, after which it rapidly decreases (7). Similarly, in this study, in both the intervention and control groups, increased lymphocyte counts were confirmed immediately after exercise completion, and approximately 2 h later, the counts markedly decreased. As there were no significant differences in these counts between the intervention and control groups, it can be assumed that the subjects in both groups had exercised with similar high intensities, and/or that the intervention had little effect.

### The effect of HBO treatment on VAS scores for whole-body and leg fatigue

With regard to fatigue-related VAS scores, in this study, although there was significant pre- to post-intervention improvement in whole-body fatigue in the HBO group, no such difference was found in the air group. In addition, the comparison of the degree of improvement between the groups showed that the improvement was significantly greater in the HBO group. The leg fatigue scores in this group significantly improved between test 2 post-exercise and test 3 post-intervention from 49.9 ± 18.4 to 26.1 ± 16.9 (*p* < 0.001), as did the scores for the air group from 54.7 ± 17.1 to 43.6 ± 24.0 (*p* < 0.05). However, the improvement was greater in the HBO group.

Akarsu et al. documented the efficacy of HBO treatment on chronic fatigue syndrome, evaluated using the VAS, Fatigue Severity Scale, and Fatigue Quality of Life Score, and found that HBO decreased the severity of symptoms and increased quality of life (25). In a study on the effectiveness of HBO treatment for acute ankle sprain, Yagishita et al. found that the VAS scores for pain decreased from pre- to post-intervention (26).

In a study on the effects of HBO on exercise-induced fatigue, Shimoda et al. documented the effect of HBO on a muscle fatigue test involving 50 isometric repetitive ankle plantar flexion exercises, reporting that HBO lessened muscle power reduction during repetitive exercise (27).

In contrast, a study that measured grip contractile force in subjects in a hyperbaric chamber found that while contractile force increased with HBO, the decline in force production was faster compared with the control group, as muscle fatigue increased (28). Furthermore, other studies have concluded that exposure to HBO before exercise does not result in improved performance (13, 21). In addition, according to the Cochrane Review, data from trials on HBO treatment for DOMS showed higher pain scores 48 and 72 h after treatment (5).

In the present study, no significant differences in exercise load were found between the HBO and air groups. However, the VAS score results for leg fatigue after the intervention were significantly lower in the HBO group than in the air group. This suggests that HBO treatment after moderate-intensity exercise may have an effect on the brain or skeletal muscles.

### Blood test results

Biochemical testing for CK serum level may have a role in monitoring healthy muscle response to training (8). In this study, although CK levels increased by approximately 10% in both the HBO and MHA groups after the workout, because testing for CK isozymes was not conducted, it was impossible to determine whether the CK had originated from skeletal muscles. The myoglobin level peaks immediately after exercise when training has been so intense that it causes exercise-induced muscle damage and returns to its pre-exercise level within 24 h (29). As such, it is also a potential muscle damage marker (18). In our results, although no increase in myoglobin levels was found post-exercise in test 2, increased levels were found in tests 3 and 4, but there was no significant difference between the groups.

Several previous studies have suggested that HBO has an effect on the immune system response. Studies have shown that HBO may inhibit immune system responses (30, 31). Hou et al. investigated the effects on TNF-α and IL-6 in patients with brain tumors and reported that the serum TNF-α and IL-6 levels were significantly lower after HBO than those in the control group (30). Oyaizu et al. reported that HBO suppressed the increase in circulating macrophages in the acute phase and then accelerated macrophage invasion into the contused muscle (31). As a result, we examined measures for numerous markers of immune system activity. However, we found no significant differences between the HBO and air groups in markers of immune system activity, including neutrophils, lymphocytes, TNF-α, and IL-6.

Oyaizu et al. found that in a rat skeletal muscle injury model treated with HBO, at 6 h and 24 h after injury, neutrophil markers had decreased, and, at 3, 5, and 7 days after injury, macrophage movement into injured tissue was accelerated (31). While this may suggest that exposure to HBO accelerates a shift in the immune system through the early mobilization of macrophages, variation in the data values was possible because of the measurement timing.

Balestra et al. analyzed microparticles (MPs) expressing proteins, including platelets (CD41), neutrophils (CD66b), endothelial cells (CD146), microglia (TMEM), and phalloidin binding and thrombospondin-1 (TSP) under six different oxygen concentrations ranging from hypoxia to hyperbaric hyperoxia. The responses were found to be different and sometimes contrasting (32, 33). MacLaughlin et al. reported that 100% normobaric oxygen mobilizes stem cells and upregulates the expression of the inflammatory cytokines of the macrophage migration inhibitory factor (MIF) and a proliferation-inducing ligand (APRIL) (34).

It would also be interesting to investigate biomarkers of inflammatory cytokines such as CD41, CD66b, CD146, TMEM, TSP, MIF, and APRIL in the future. In addition, it would be desirable to increase the sample size and conduct longer follow-up studies, and additional testing items may potentially allow for an objective evaluation of subjective efficacy.

Balestra et al. proposed the “normobaric oxygen paradox,” a phenomenon whereby tissues sense a return to normoxia after a hyperoxic event as oxygen shortage, resulting in the upregulation of hypoxia-inducible factor 1 (HIF-1) transcription factor activity. It has been reported that differences in oxygen concentration, such as hyperoxia and hypoxia conditions, and changes in oxygen concentration from hyperoxia to normobaric oxygen, affect the inflammatory response (35, 36). Therefore, it may be beneficial to conduct future studies that compare not only HBO but also 100% oxygen administration and examine the effects of the impact of different oxygen concentrations and mild hyperbaric air itself as a control group.

Other recent studies have suggested that HBO mobilizes stem cells (37, 38), and exposure to HBO after moderate-intensity training may function protectively by mobilizing stem cells to damaged muscle.

In this study, the subjects' self-ratings indicated that HBO treatment may be effective in promoting recovery from fatigue after long-duration, moderate-intensity exercise. However, this was not objectively supported by the blood test results.

Further detailed studies are needed to understand the mechanisms of the effects of oxygen or hyperbaric oxygen on fatigue and recovery.

### Study limitations

The limitations of this study include the small sample size, the limited number of biomarkers used, and the fact that the measurements were only performed 1.5 h and 24 h after the intervention. In addition, this study only considered moderate-intensity exercise. Regarding the generalizability of these findings, we can only suggest the possibility of HBO effects. If the measurements are performed at different times and/or with additional blood test items, the results may be more definitive. Future studies need to evaluate the effects of HBO treatment after other types of intense exercises, such as short-duration, high-intensity exercise.

## Conclusion

In this study, the subjects' self-ratings indicated that HBO treatment may be effective in promoting recovery from fatigue after long-duration, moderate-intensity exercise. However, this was not objectively supported by the blood test results. We had hypothesized that HBO would reduce fatigue, but the results of this study could not determine the efficacy of HBO on exercise-induced fatigue. Consequently, at present, the use of HBO to hasten recovery from exercise-induced fatigue cannot be recommended.

However, because the subjective evaluations of whole-body fatigue improved in this blinded trial, HBO may have some positive effects on recovery from exercise-induced fatigue via the central nervous system or skeletal muscles. More studies are needed to clarify the effect of HBO on fatigue.

## Data Availability

The raw data supporting the conclusions of this article will be made available by the authors, without undue reservation.
